# Left sided Amyand hernia – A case report

**DOI:** 10.1016/j.ijscr.2022.107374

**Published:** 2022-07-01

**Authors:** Jayateertha Joshi, Esha Mallik, Talha Ahmed, Rahul Bhat, George M. Varghese

**Affiliations:** a Department of General Surgery, Kasturba Medical College, Mangalore, Manipal Academy of Higher Education, Manipal, India; b Kasturba Medical College, Manipal, Manipal Academy of Higher Education, Manipal, India

**Keywords:** Amyand hernia, Inguinal hernia, Appendix, Case report

## Abstract

**Introduction:**

Amyand hernia is a clinical condition wherein content of the inguinal hernial sac is formed by the vermiform appendix.

**Case presentation:**

1 year 3-month-old male child presented to our OPD with an irreducible left inguinal hernia for which he was taken up for an emergency herniotomy. The terminal ileum, caecum and appendix were found to be the contents of the hernial sac.

**Discussion:**

As the appendix is anatomically located on the right, Amyand hernia more commonly occurs on the right, however its occurrence on the left, is a rare event and is usually associated with congenital anomalies like Intestinal malrotation, Situs inversus and mobile caecum.

**Conclusion:**

Amyand hernia presenting on the left is extremely rare and high index of clinical suspicion is required to manage such patients. We report one such rare case of a Left sided Amyand hernia in a young child.

## Highlights

• Amyand hernia is the presence of appendix in the hernial sac.
• Although more common on the right, it may rarely present on the left side.
• Situs invertus, Malrotation, and Mobile caecum are common causes of left sided Amyand hernia and thus need to be ruled out.
• High index of clinical suspicion is required as a diagnosis of Amyand hernia is generally made intra-operatively.
• Standard management is based on Losanoff and Basson’s criteria, however it can be individualized as seen in our case.

## 1 Introduction

A French Surgeon – Claudius Amyand described this now eponymous hernia in 1735; Amyand hernia is a condition where the appendix is seen as a content within the sac of an inguinal hernia. The appendix may be inflamed/perforated, or it may be non-inflamed in an irreducible hernia and is a rare entity, with an incidence of 0.1 % and 1 % respectively [1], [2], [3]. He initially described a perforated appendix in the hernial sac of an eleven year old male child who had come with a right sided inguinal hernia and a faecal fistula [2]. As the appendix's normal anatomical position in on the right within the peritoneum, Amyand hernia occurs more commonly on the right side than the left. This may also be due to the more common occurrence of right sided inguinal hernias, as opposed to left [1], [2]. Having similar presenting features as that of an incarcerated or strangulated hernia and acute appendicitis, Amyand hernia is a difficult condition to diagnose pre-operatively, and hence is generally diagnosed intraoperatively [4].

We describe one such case of a rare occurrence of left sided Amyand hernia in a male child aged 1 year 3 months is reported. This case report has been reported in line with the SCARE Criteria [5].

## 2 Case description

`

A male child, aged 1 year and 3 months, was brought by his mother to the Paediatric Surgery outpatient department (OPD) in Mangalore, Karnataka, India. She complained of noticing a swelling in the left inguino-scrotal region of the child for 1 year. The mother gave history of the swelling being small and localized to the left inguinal region initially, which reduced spontaneously, and reappeared when the child cried. However, over the last 2 days, the mother noticed that the swelling had increased in size and now involved the left inguino-scrotal region, with the swelling not reducing by itself. The child had no complaints of feeding difficulty, abdominal distension, vomiting and bowel or bladder complaints. There were no similar complaints on the right side. The child was born at 39 weeks by a constitutional normal vaginal delivery. There were no complications encountered during delivery and the immediate post delivery period. The child had no jaundice in the immediate neonatal period and was started on breast feeds with kangaroo care. The child has been appropriately immunized for age and has no delay in developmental milestones. This was the 1st child borne by the mother and there is no significant family history.

**Fig. 1:**
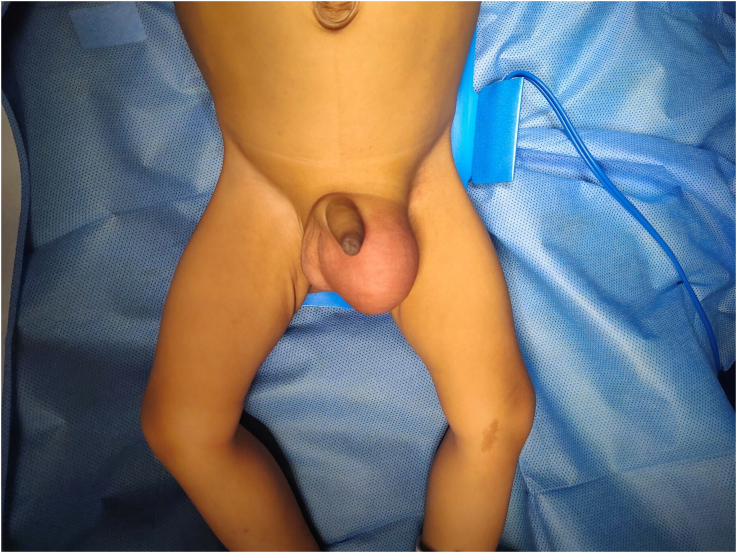
Left inguino-scrotal swelling as seen on inspection, which was found to be irreducible on palpation.

On inspection there was a left inguinal scrotal swelling noted measuring 4x3cm extending to the root of the scrotum. The swelling was irreducible, with no expansile impulse noted when the child cried. On palpation of the hernia, there was no localized tenderness or warmth. The hernia was seen extending from the superficial inguinal ring to the root of the scrotum and was irreducible with gurgling sounds present, suggestive of enterocoele. Getting above the swelling was not possible. The left testis was palpated separately from the hernial sac. Right hernial orifices were free and right testis was in normal position. Rest of the abdomen examination revealed no organomegaly or free fluid in abdomen. Examination of the external genitalia was normal. At the end of the clinical examination, a diagnosis of left complete irreducible inguinal hernia was made with enterocoele as the content (Fig. 1). Respiratory and Cardiovascular system examinations were within normal limits. There were no deformities noted on Musculoskeletal examination.

## 3 Diagnostic assessment

Baseline blood investigations necessary for anesthetic clearance were obtained. Chest X-ray done was within normal limits. As the child presented with left complete irreducible inguinal hernia, it was deemed to be a surgical emergency and hence the child was directly taken up for left inguino-scrotal exploration.

## 4 Therapeutic intervention

As the child was clinically diagnosed to have an irreducible inguinal hernia, he was taken up for an emergency left inguinal exploration with hernia repair under general anesthesia. The complications of the procedure including need for formal laparotomy, resection, and anastomoses in case of gangrenous bowel, recurrence of hernia at a later age, inguinodynia, injury to vas deferens including possibility of infertility, injury to testicular vessels, hemorrhage, wound infection was thoroughly conveyed to the parents and written consent was obtained.

The procedure was carried out by a senior paediatric surgeon in our tertiary hospital who has a surgical experience of over 8 years. The procedure was also performed under the care of a specialist paediatric anesthetist with an experience of over 15 years.

**Fig. 2:**
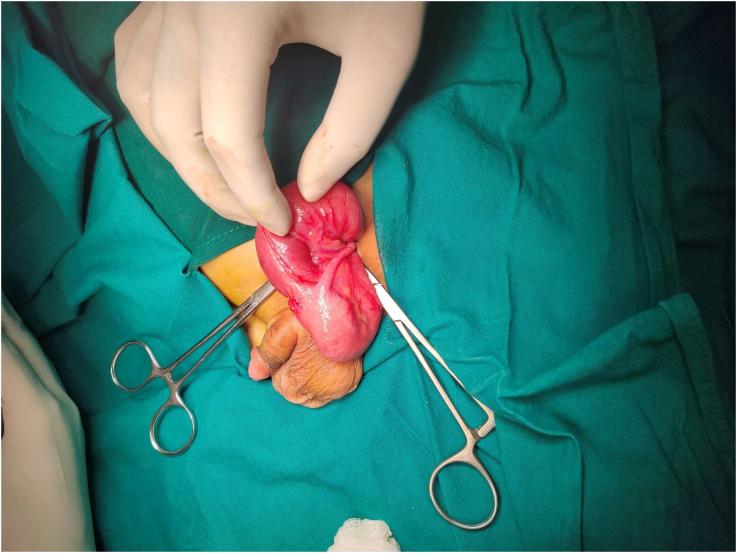
Terminal ileum, caecum and appendix seen as the contents of the hernial sac, following inguino-scrotal exploration.

Left groin crease skin incision was given 1 in. proximal and parallel to the inguinal ligament measuring approximately 4 cm in length. After dissecting the subcutaneous fascia, the external oblique aponeurosis was visualized and opened along the fibers angled towards the superficial ring. Ilioinguinal nerve was safeguarded, and the sac was visualized. The cord structures were secured and placed in a hernial ring. Left indirect sac was noted arising from the deep ring and situated lateral to the inferior epigastric pulsations. The sac was carefully dissected off the cord structures. Once the sac was skeletonized, the indirect sac was opened at the summit and the terminal ileum, caecum and the appendix were found to be the contents. None of the contents of the sac were inflamed or strangulated (Fig. 2). Hence, to avoid contaminating the field, and complicating an otherwise aseptic procedure, appendectomy was not done. Moreover, due to a high degree of suspicion of intestinal malrotation, which if present would have necessitated re-exploration and Ladd's procedure, an on-table decision was made against performing appendectomy during the initial surgical procedure.

Contents were reduced and herniotomy was done by ligating the sac close to the deep ring using 3–0 absorbable polyglactin sutures and excising the excess sac, after safeguarding the cord structures. There was no visible bulge noted in the Hasselbach's triangle hence ruling out a direct component. External oblique aponeurosis was closed using continuous 3–0 absorbable polyglactin sutures, followed by skin closure using non-absorbable 3–0 nylon sutures.

## 5 Follow-up and outcomes

**Fig. 3:**
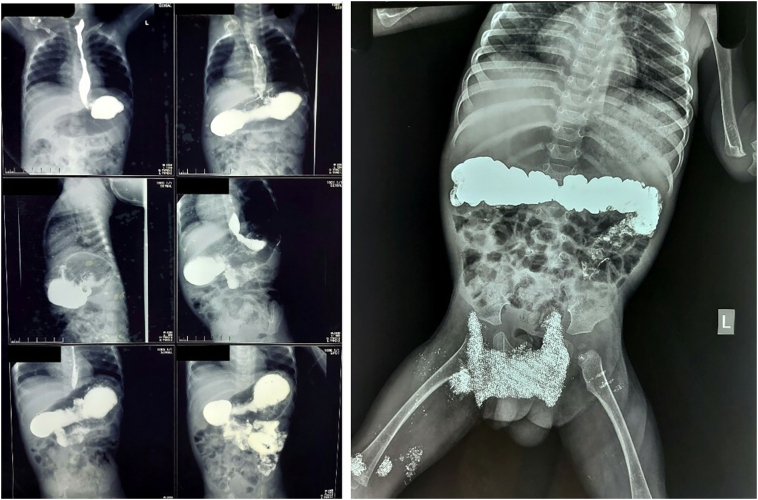
Normal barium meal follow through study, ruling out intestinal midgut malrotation.

As appendix was the content of the left sided inguinal hernia, a diagnosis of left Amyand Hernia was made. The child was subjected to a barium meal follow-through on post-op day 4 to ascertain the cause. As the child had presented with a surgical emergency, the same was not done pre-operatively. As depicted in the figure below (Fig. 3), there was no evidence of situs inversus or intestinal malrotation; and hence mobile caecum was the likely underlying cause for this left sided Amyand Hernia.

The post-operative period was uneventful and the child was initiated on breast feeds from post-operative day 1. His bowel habits had recovered and there was no evidence of infection at the surgical site, hence patient was discharged on post-op day 4. The child came for suture removal on post-op day 10, which showed a completely healed scar.

He was followed-up regularly for a 6-month period which showed no further recurrence of the hernia.

## 6 Discussion

When the appendix forms the content of the inguinal hernial sac, such a clinical condition is eponymously named as Amyand hernia. It may present as a non-inflamed appendix in an irreducible sac, as seen in 1 % of the cases, or it may have a much rarer presentation with appendicitis which is only seen in 0.1 % of the cases [1], [2].

As the vermiform appendix has its normal anatomical position on the right side of the body, this condition is more common on the right as compared to the left. The more common occurrence of an inguinal hernia on the right side may also have a role to play in this. The presence of an Amyand hernia on the left side is relatively rarer and may be seen as a consequence of [1], [2]:

▪ Situs Invertus
▪ Intestinal Malrotation
▪ Mobile Caecum

Amyand hernia may occasionally mimic a complicated inguinal hernia, acute appendicitis or both, in its presentation. Moreover, in cases where Amyand hernia presents on the left side, there is generally no tenderness at McBurney's point due to the unusual anatomy, thus making it difficult to rule out acute appendicitis [3]. While the exact pathophysiology leading to an inflamed appendix within the hernial sac remains unknown, it has been proposed by Weber et al., that the process of herniation leads to an increased vulnerability of the appendix to repeated micro-trauma, which in turn causes fibrosis, thus leading to the vermiform appendix becoming adherent to the hernial sac [6]. Furthermore, this hypothesis of an inflamed appendix leading to incarceration, resulting in decreased vascularity and an increased growth of bacterial flora has been supported in literature by several authors [7], [8], [9]. Abu-Dalu J, et al. have also proposed the possibility of sudden muscular contraction or an increased abdominal pressure being the cause of appendiceal compression [7], [10], [11].

Although a CT scan may be useful in detecting an Amyand hernia, as indicated by a systematic review of literature by D. Papaconstantinou, et al. [12]; it is not the imaging modality of choice in case of uncomplicated inguinal hernias and a pre-operative diagnosis via sonography is highly reliant on the experience and proficiency of the operator, thus rendering it a relatively unreliable method. Due to these reasons, the likelihood of reaching a diagnosis of an Amyand hernia pre-operatively is rare, and it is usually diagnosed intraoperatively [4]. Furthermore, in case of a left sided Amyand hernia, post-operative imaging procedure is advised to determine the cause for the left sided occurrence of the hernia [2].

This condition has been classified into 4 subtypes depending upon the clinical presentation of the patient and the status of the appendix, where Type 1 refers to an Amyand hernia with no inflammatory changes, Type 2 refers to Amyand hernia with septic changes restricted to the hernial sac, Type 3 refers to Amyand hernia involving the spread of sepsis to outside the hernial sac and Type 4 refers to Amyand hernia with acute appendicitis and other related or unrelated abdominal lesions [13].

Based on this classification, a criterion was devised (Losanoff and Basson's [2]) to ascertain the plan of surgical management wherein the first type is managed simply by reducing the hernia and inserting a mesh; and in young patients, appendectomy may be done. In case of type 2 Amyand hernia, and appendectomy is done through the hernia along with a primary repair of hernia. In this case, no mesh is inserted. Type 3 is managed similar to type 2, however in this case, a laparotomy is done along with an appendectomy and primary hernia repair. Management of type 4 is similar to type 1–3 hernia management, along with appropriate treatment of the second pathology.

While the Losanoff and Basson's criteria provides a standardized approach in the management of Amyand hernia, the treatment plan for the same, particularly that of Type 1 Amyand hernia, remains controversial, with surgeons often making a call against performing a prophylactic appendectomy in case of the presence of a non-inflamed vermiform appendix within the sac, to avoid complicating an otherwise aseptic surgical procedure [4]. Additionally, there is evidence in literature that the vermiform appendix may play a useful role in modulating the immunity of the gut, especially in children, and a missing appendix may lead to an increased incidence of gastrointestinal illnesses [4], [14]. Others are of the opinion that a prophylactic appendectomy, particularly in cases of a left sided Amyand hernia, is necessitated as,

• There is a high risk of recurrence of hernia, and
• Any case of appendicitis in the future may present with atypical features [1], [4]

In our case, the child had a Type 1 Amyand Hernia as per the Losanoff and Basson classification. However, with regards to management, based on the intra-operative findings, an on-table decision was taken to act independent of the classification and to proceed with reduction of hernial contents and perform herniotomy alone as the appendix was found to be normal and uninflamed within the sac, and none of the other contents were gangrenous or ischemic. Moreover, as the risk of mesh infection in cases presenting with irreducibility are considerably higher [3], the operating team decided against a mesh repair in our patient. As there was no situs invertus or intestinal malrotation observed on imaging in our patient, a mobile caecum was the likely cause for the left sided nature of the hernia in this case.

## 7 Conclusion

Amyand Hernia is a rare clinical condition wherein the vermiform appendix is part of the sac content in an inguinal hernia. Typically presenting on the right side due to the normal anatomical position of the appendix on the right, left sided occurrences have also been documented, usually caused by an underlying embryological pathology affecting the gut location and mobility. Despite CT and Ultrasonography being good aids for diagnosis for this condition, a definitive diagnosis can only be made intra-operatively. We have reported a similar case of a very rare left sided Amyand hernia in a 1 year 3-month-old male child who presented with an inguino-scrotal swelling with irreducibility of contents. The child was managed with herniotomy alone. As there was no situs invertus or intestinal malrotation observed on further imaging; a mobile caecum was the likely underlying cause in this case. Regular follow-ups showed no further recurrence of the hernia. The management in such a condition must be individualized, with a high index of clinical suspicion needed while operating on such patients.

## Informed consent

Written informed consent was obtained from the parents of the patient for publication of this case report and accompanying images. A copy of the written consent is available for review by the Editor-in-Chief of this journal on request.

## Sources of funding

None.

## Ethical approval

Ethical approval was received from the Institutional Ethics Committee of Kasturba Medical College, Mangalore (Reg. no. ECR/541/Inst/KA/2014/RR-17).

## Research registration

N/A.

## Guarantor

Dr. Jayateertha Joshi is the guarantor.

## Provenance and peer review

Not commissioned, externally peer-reviewed.

## CRediT authorship contribution statement

Dr. Jayateertha Joshi – Reviewing and Editing the article, Surgical operation, Dr. Esha Mallik – Study concept, Study design, Data collection, writing of original draft, Surgical Operation, Dr. Talha Ahmed – Study concept, writing and editing of article, Dr. Rahul Bhat – Reviewing and final approval of the version to be submitted, Dr. George M Varghese - Study concept, writing and editing of article.

## Declaration of competing interest

None.
